# Can long-term care insurance reduce catastrophic health and long-term care expenditures among older adults? A quasi-experimental study in China

**DOI:** 10.1007/s10433-025-00861-1

**Published:** 2025-06-03

**Authors:** Chengxu Long, Wei Yang, Karen Glaser

**Affiliations:** Department of Global Health and Social Medicine, King’s College London, London, England, UK

**Keywords:** Catastrophic expenditure, Long-term care costs, Cognitive impairments, Physical limitations, Long-term care insurance

## Abstract

Older adults with cognitive and/or physical limitations often face excessive health and long-term care (LTC) costs, which significantly affect their living standards. However, current measures of catastrophic health expenditure primarily focus on healthcare costs, overlooking the substantial burden of LTC costs. Our study is among the first to introduce the concept of catastrophic health and long-term care expenditures (CHLTCE) to comprehensively capture the economic burden of health and LTC costs associated with functional limitations. Drawing data from 2011, 2013, 2015, and 2018 waves of China Health and Retirement Longitudinal Study, we employed a staggered difference-in-differences approach to assess the impact of long-term care insurance (LTCI) on alleviating CHLTCE. We found that after accounting for LTC costs, both total health and LTC expenditures and CHLTCE risk significantly increased compared to when only healthcare costs were considered. Furthermore, LTCI coverage significantly reduced CHLTCE risks among older adults with cognitive and/or physical limitations. This effect was more pronounced among those with severe cognitive impairments and those with physical limitations and chronic diseases. In addition, its effectiveness in reducing CHLTCE risks for individuals with complex care needs was observed only at the high threshold, but not at lower thresholds. Our findings offer empirical contributions by incorporating both health and LTC costs into catastrophic expenditures analysis. We recommend that policymakers refine LTCI eligibility criteria and implement tiered coverage options based on various levels and dimensions of cognitive and physical function—beyond activities of daily living—to offer targeted protection for individuals with complex care needs.

**Supplementary Information:**

The online version contains supplementary material available at 10.1007/s10433-025-00861-1.

## Introduction

Globally, the number of people with cognitive impairments (CI) in 2023 is around 55 million (World Health Organization 2021). Impaired cognition significantly impacts individuals’ physical health and abilities to perform activities of daily living (ADLs), resulting in costly healthcare and long-term care (LTC) needs (Hurd et al. 2013). Catastrophic healthcare expenditure is widely used to capture the effect of health expenditures on family living standards (Li et al. 2022). However, this indicator often underestimates the financial impact associated with receiving LTC when estimating the economic burden among older adults with cognitive and/or physical limitations, as these individuals are frequent users of both health and LTC services (Li et al. 2022).

This study is one of the first studies to introduce the concept of Catastrophic Health and Long-term Care Expenditure (CHLTCE), which refers to both health and LTC costs that exceed a certain percentage of a household’s income (Del Pozo-Rubio et al. 2019). The conventional method for calculating catastrophic healthcare expenditures among individuals with CI primarily considers direct out-of-pocket expenses linked to healthcare services (Li et al. 2022). Yet, this technique frequently neglects LTC expenses tied to CI, which can substantially impact the living standards of households. Although several studies have examined LTC costs in the context of catastrophic expenditures among older adults with physical limitations (Del Pozo-Rubio and Jimenez-Rubio 2019; Del Pozo-Rubio et al. 2019), they have not accounted for healthcare expenditures and have excluded older adults with CI who often incur substantial LTC costs. Our definition captures not only healthcare costs but also LTC costs, i.e., costs associated with paid non-medical services for individuals requiring assistance with ADLs, as understanding these costs is crucial for assessing how households are impacted by caring for older adults with cognitive and/or physical limitations.

Another unique aspect of this study is the inclusion of costs related to the provision of informal care in our analysis. Research indicates that informal caregivers, typically those who provide care without compensation, account for 40–75% of the total care received by individuals with severe cognitive impairments (Angeles et al. 2021). Despite the fact that family members may sacrifice work hours or jeopardize their career prospects to fulfill caring responsibilities, the significance of informal care and its cost implications in CHLTCE are often overlooked in previous studies. Therefore, our measure of LTC expenses takes into account informal care costs.

Scholars have been advocating for the implementation of effective LTC policies to alleviate the financial difficulties of older adults in need of care (Lei et al. 2022). One such policy is long-term care insurance (LTCI), which offers coverage for services that assist older adults with daily living activities (Kwak et al. 2021). Evidence suggests that LTCI can help reduce out-of-pocket expenditures for LTC services (Lei et al. 2022). It is also suggested that LTCI may significantly decrease healthcare costs by contributing to health improvements, as professional carers may better manage the health conditions of disabled older adults, potentially reducing their reliance on healthcare services (Feng et al. 2020). Furthermore, LTCI is likely to alleviate the care burdens faced by informal carers by substituting informal care with formal LTC (Lei et al. 2022). This, in turn, can enhance the participation of informal carers in the labor market and subsequently improve household financial status (Pei et al. 2024). However, previous studies have not examined the effects of LTCI on catastrophic expenditures among older adults in China.

We chose China as a case study to investigate the influence of LTCI on CHLTCE among older adults with cognitive and/or physical limitations. In 2018, approximately one out of every five (20.8%) Chinese people aged 65 years and above had CI (Jia et al. 2020). Since China’s LTCI covers the expenses for basic daily assistance and associated health and nursing care for eligible individuals with critical LTC needs (Zhou and Dai 2021), older adults with CI—especially those with severe CI—are likely to benefit from LTCI, given their frequent difficulties in performing basic daily activities (Hurd et al. 2013). Therefore, LTCI may have the potential to reduce catastrophic expenditures for older adults with cognitive and/or physical limitations. What remains to be understood is the extent of LTCI’s effect on reducing CHLTCE and how this effect varies by the levels of cognitive and physical limitations, given that LTCI reimbursement is primarily determined by the degree of physical disability and CI (Hao and Li 2021).

Drawing on data from the China Health and Retirement Longitudinal Study (CHARLS) 2011, 2013, 2015, and 2018, this paper aimed to examine the impact of LTCI on CHLTCE among older adults with cognitive and/or physical limitations, and whether this effect varies by cognitive and physical function. Specially, we sought to answer the below research questions:

1. Can LTCI help reduce the CHLTCE risk among older adults with cognitive and/or physical limitations?
2. Does the influence of LTCI on reducing CHLTCE risk vary by cognitive and physical function?

## Literature review

### Measuring health and long-term care costs in catastrophic health and long-term care expenditure

As previous, previous studies have not considered healthcare, formal care, and informal care costs together when calculating catastrophic expenditures among older adults with cognitive and/or physical limitations. To address this gap, we introduce the concept of CHLTCE, which includes not only healthcare costs but also formal and informal care costs. The following section will discuss each cost category and emphasize the importance of incorporating them in estimates of CHLTCE.

The first cost category—out-of-pocket healthcare costs—involves fees for consultations, examinations, prescriptions, and other pertinent charges levied by healthcare providers throughout the treatment process (Yang and Hu 2022). Research has suggested that healthcare expenditures among older adults with cognitive and/or physical limitations are substantial (Deardorff et al. 2019; Reichard et al. 2011). It is estimated that Americans with CI and those with physical limitations had average annual healthcare expenditures of $11,487 and $10,288, respectively, which were 4.8 and 4.3 times higher than the expenditures of individuals without such limitations (Reichard et al. 2011). For individuals with severe functional limitations, these costs may be even higher. A study conducted in the USA showed that older adults with severe CI can expect to incur $20,148 more in annual healthcare costs compared to those without (Deardorff et al. 2019). Another US study reports that severe disability increases the probability of spending 30% points more of total healthcare costs (Lukemeyer et al. 2000).

The second cost category is the out-of-pocket formal LTC costs. Typically, LTC costs take two forms: costs for formal LTC services and costs for informal caring (Hurd et al. 2013). Formal LTC can include institutional care (e.g., nursing homes), community-based care, or care from paid helpers (Hurd et al. 2013). Formal care expenses for individuals with cognitive and/or physical limitations can be substantial (Hurd et al. 2013), placing them at a greater risk of incurring CHLTCE than the general population. Evidence shows that the average LTC costs associated with moderate and severe physical limitations were $1,722 and $52,555, respectively (Mitra et al. 2017). Evidence indicates that globally formal care costs among individuals with severe CI constitute 40% of all costs (Prince et al. 2015). Researchers have also found that individuals with severe CI are likely to face an additional $28,500 in annual formal care costs compared to the healthy population in the USA (Hurd et al. 2013).

The third cost category consists of costs associated with informal care provision. This category is often underestimated, despite informal care being the primary source of care for individuals with CI in many countries (Li et al. 2022). Informal carers frequently dedicate a significant amount of their time to providing care (Kwak et al. 2021). Some may need to sacrifice employment and associated income to care for disabled older adults (Van den Berg et al. 2006), resulting in missed career opportunities and a reduction in household income (Van den Berg et al. 2006). It is estimated that providing informal care for more than 10 h per week results in an 18% and 9% reduction in wages for men and women in the UK, respectively (Carmichael and Charles 2003). Research has also revealed that the costs of informal care for individuals with severe CI account for about 40% of total expenses in high-income countries and 66% in low- and middle-income countries (Wimo et al. 2013). Therefore, including informal care costs in the analysis is crucial for understanding the economic challenges faced by older adults with cognitive and/or physical limitations as they often require intensive informal care support.

Despite this importance, few studies have accounted for LTC costs, particularly those related to informal care, when assessing catastrophic expenditures among individuals with cognitive and/or physical limitations. There is an urgent need to enhance our understanding of how these costs impact household living standards.

### Impact of long-term care insurance on reducing health and long-term care costs

We situate this study in the context of China and evaluate the impact of LTCI on reducing CHLTCE. Existing studies on LTCI have largely found positive effects on reducing health and/or LTC expenses (Hurd et al. 2013; Kim and Lim 2015). LTCI can directly lower the recipient’s out-of-pocket costs by reimbursing formal care expenses (Zhu and Oesterle 2019). In addition, by improving access to professional care services, LTCI can reduce the care burden on informal caregivers (Pei et al. 2024). Specifically, through financial protection via reimbursements, LTCI may reduce the number of informal care hours provided by family caregivers (Pei et al. 2024). This reduction in caregiving hours can enable family caregivers to return to the workforce, potentially improving household income and overall financial conditions (Pei et al. 2024). Furthermore, by improving access to LTC services, LTCI may enable individuals to receive the necessary care promptly, thereby decreasing unmet care needs and contributing to better health outcomes. Accordingly, this could lead to reduced use of additional healthcare services and lower overall healthcare expenses (Feng et al. 2020).

In China, to ensure that older disabled adults can access affordable care services, the Chinese government initiated national-level LTCI pilots in 15 cities and two provinces (Jilin and Shandong) beginning in 2016 (Lei et al. 2022). In the LTCI pilots, six cities have extended LTCI coverage to older adults with CI (Zhou and Yuan 2021). In the remaining pilot cities, older adults with CI can also benefit from LTCI if they have critical LTC needs (Zhou and Yuan 2021). The eligibility for LTCI hinges on the assessment of LTC needs, primarily using the Barthel ADL scale and/or cognitive scales, which measure independence in performing ADLs or instrumental activities of daily living (IADLs) (Zhou and Dai 2021). A significant portion of older adults with CI, especially those with severe CI, often have difficulties in performing ADLs/IADLs and critical long-term care needs (Perneczky et al. 2006), making them highly likely to qualify for LTCI benefits. Specifically, LTCI primarily provides three types of services for eligible individuals: (1) hospital care at designated hospitals providing 24-h care, (2) nursing home care provided by designated caregivers, and (3) home-based help, which includes regular visits from home care assistants (Yang et al. 2021). The key features of LTCI in China can be found in Supplement A.

Although previous studies have shown that Chinese LTCI played a role in reducing institutional care use and healthcare expenses (Feng et al. 2020; Lei et al. 2022), they have not focused on older adults with CI. It is also unclear whether China’s LTCI pilot schemes can alleviate CHLTCE among this population. In addition, some LTCI pilot cities (e.g., Chengdu) cover individuals with critical physical limitations or severe CI but exclude those with mild CI (Hao and Li 2021). Therefore, the effects of LTCI on CHLTCE may vary by the level of cognitive and physical limitations. Furthermore, evidence suggests that certain chronic diseases, such as stroke, are strongly associated with physical limitations (Hung et al. 2012). Research also indicates that individuals with disabilities have a higher risk of developing chronic diseases (World Health Organization 2024a). Based on the care level of beneficiaries, LTCI offers increased reimbursement for care services (Zhou and Dai 2021). In this regard, the effects of LTCI on CHLTCE may vary by chronic diseases and physical limitations.

## Materials and methods

### Data

We employed four waves of data (2011, 2013, 2015, and 2018) from CHARLS. CHARLS is a national longitudinal survey initiated in 2011, targeting individuals aged 45 and older, and follows a multi-stage, stratified probability sampling design (Kwak et al. 2021). To rule out the impact of COVID-19, we excluded the 2020 wave of CHARLS from our analysis. CHARLS offers measurements on cognition, physical function, healthcare expenditures, and LTC provision, which align well with our study aim.

The analytical sample consists of 31,076 observations from 9312 Chinese individuals aged 60 and above with cognitive impairments (defined as a cognitive summary score < six (Jak et al. 2009; Li et al. 2022)) or physical limitations at baseline. Since the questions measuring cognition at each wave of CHARLS are inconsistent, we followed previous research (Jak et al. 2009; Li et al. 2022) and calculated cognitive function by summing the number of correct responses to the 11 questions consistently asked in all waves. The total score ranges from 0 to 11, with a higher score indicating better cognitive function. Consistent with previous studies (Jak et al. 2009; Li et al. 2022), the respondent was categorized as having cognitive impairments if the summary score was less than six. Besides, we followed previous research (Hu et al. 2022) and used ‘at least one difficulty with ADLs, IADLs, and mobility tasks’ as an indicator of physical limitations. Details of the measurement of cognitive impairments and physical limitations can be found in Supplement B. Supplement C shows the flowchart of the sample.

### Variable specifications

#### Dependent variable

The outcome variable is the likelihood of experiencing CHLTCE. These indicators were calculated using measures of total health and LTC costs by aggregating out-of-pocket healthcare expenditures, out-of-pocket formal care costs, and informal care costs. We calculated the proportion of an individual’s health and LTC costs relative to their annual household expenditures (details can be found in Supplement Section D). An individual is marked as incurring CHLTCE if $\frac{{H}_{i}}{{E}_{i}}>{z}_{l}$, where ‘${H}_{i}$’ is out-of-pocket health and LTC costs of the individual i and ‘${E}_{i}$’ refers to the household expenditures (World Health Organization 2024b). According to WHO and previous research (World Health Organization 2024b; Yang and Hu 2022), ‘${Z}_{l}$’ represents predetermined thresholds, defined as 20% and 25% of total household expenditures, along with 40% of non-food household expenditures. We did not adopt the 10% of total household expenditure threshold in the main analysis, as previous research suggests that a low threshold may overestimate catastrophic expenditures (Arsenijevic et al. 2013). Specifically, we created three sets of dichotomous variables indicating whether the proportion of out-of-pocket health and LTC costs exceeded the defined threshold in each survey wave.

#### Independent variable

The independent variable in our study is whether individuals were covered by LTCI at the time of the survey waves (Pei et al. 2024). In CHARLS, it is possible to identify respondents’ municipality of residence. Among the 15 initial national-level LTCI pilot cities, only 12 were included in our study sample. Among the two pilot provinces, four cities have also initiated the LTCI, which were also included in our sample. Therefore, there are 16 pilot cities in our study sample, and the eligibility criteria are closely associated with individuals’ Social Health Insurance (SHI) status (see Supplement Table E1) (Pei et al. 2024). Following the approach used in previous research (Lei et al. 2022; Pei et al. 2024), we defined the treatment group as individuals residing in the 16 pilot cities who were eligible for LTCI, and the control group as those residing in non-pilot cities. Details on the identification of these two groups are provided in Supplement Section E.

**Table 1:** Weighted descriptive statistics of 31,076 observations

	2011, Mean (SD)		2013, Mean (SD)		2015, Mean (SD)		2018, Mean (SD)	
	Control	Treated	Control	Treated	Control	Treated	Control	Treated
*Incurring CHLTCE (%)*								
20% of total expenditures	17.14	11.82	11.44	7.24	22.80	17.13	28.21	28.72
25% of total expenditures	16.01	10.15	10.20	4.92	21.15	15.70	27.09	28.72
40% of non-food expenditures	16.84	11.91	12.63	6.65	25.04	19.85	31.33	26.81
Total health and LTC expenditures (1,000 CNY)	6.46 (21.85)	3.89 (16.51)	5.59 (22.49)	1.47 (4.50)	20.69 (69.77)	14.18 (46.43)	35.72 (125.81)	34.18 (100.42)
Age	69.90 (7.71)	67.61 (5.56)	71.47 (7.53)	69.47 (5.70)	72.80 (7.16)	71.34 (5.68)	75.47 (6.95)	74.62 (5.65)
Female (%)	59.09	52.98	59.23	53.10	59.84	52.02	59.49	51.30
*Education (%)*								
no formal education	34.07	20.38	34.69	19.91	34.65	19.19	33.73	19.46
elementary school	38.27	42.20	38.87	40.72	38.83	39.70	38.75	38.89
middle school or above education	27.65	37.42	26.44	39.37	26.52	41.11	27.52	41.66
Married/cohabiting (%)	72.40	95.46	81.46	92.52	80.87	90.78	78.20	86.04
*Self-rated health (%)*								
Good	16.57	17.26	17.41	17.65	17.03	18.53	17.32	12.34
Fair	45.94	58.13	47.42	55.45	49.36	58.27	47.55	57.16
Bad	37.48	24.60	35.18	26.90	33.61	23.20	35.08	30.50
Number of ADL/IADL limitations	0.62 (1.74)	0.31 (1.06)	0.71 (1.71)	0.25 (1.15)	0.75 (1.72)	0.55 (1.49)	0.91 (2.01)	1.29 (2.55)
Having SHI (%)	93.00	94.17	96.08	94.55	90.43	88.71	96.46	100
Depressive symptom score	19.38 (6.45)	17.12 (6.45)	18.52 (6.09)	16.97 (5.29)	18.90 (6.69)	16.30 (5.98)	19.22 (7.12)	16.34 (7.72)
Unweighted observations	9,172	140	7,951	140	7,118	140	6,277	138

Longitudinal weight was applied

LTC, long-term care; ADL, activities of daily living, IADL; instrumental activities of daily living; SHI, Social Health Insurance

#### Control variables

Previous studies have shown CHLTCE is associated with factors such as age, gender, education level, marital status, place of residence, annual household expenditures, SHI Status, and other health measures, such as self-rated health, number of ADL/IADL limitations, and depressive symptom scores (Li et al. 2022; Yang and Hu 2022). We controlled these variables in the analyses (see Supplement F).

### Identification strategy

The analyses in our study consist of three steps (details can be found in Supplement G). First, we compared catastrophic expenditures using two measures: one solely considering out-of-pocket healthcare expenditures and the other also including formal and informal care costs. Individual longitudinal weights with household and individual response adjustments were applied to the summary statistics.

Second, to answer the first research question (RQ1), we conducted staggered difference-in-differences (DID) analyses with individual fixed effects (Sun and Abraham 2021) to analyze the impact of LTCI on CHLTCE, as follows:

$$y_{ct} = \alpha_{i} + \lambda_{t} + \sum {_{g \notin C} } \sum {_{d \ne - 1} } \beta_{g,d} (1Ei = g*D_{i} t^{d} ) + \varepsilon it$$

where *y*_*ct*_ denotes the outcome variable: the likelihood of experiencing CHLTCE, for individual _*i*_ at time _*t*_. E_i_ is the time when individual _*i*_ is initially covered by the LTCI, which is equal to “∞” for never-treated individuals. Whether an individual is covered by the LTCI or not can be determined by their eligibility rule (see Independent variable section). $g\, \in \,\left\{ {2012, \, 2015, \, 2017, \, \infty } \right\}$, indicating disjoint cohorts. We set $C\, = \,\left\{ \infty \right\}$ because there is a never-treated cohort. *d* denotes the relative time between *E*_*i*_ and _t_. ${D}_{it}^{d}$ is an indicator for individual *i* being *d* periods away from the initial treatment at time _*t*_. For the treatment groups, ${D}_{\text{it}}^{d}$=1 and individual *i* is in *d* periods away from the initial treatment at time _t_. For never-treated individuals, ${D}_{\text{it}}^{d}$=0. *β* is the estimation of the net impact of expanding LTCI coverage. Our results show that the potential trends in the outcome variables in both the treated and control groups were parallel without the implementation of LTCI. *P*-values < 0.05 were considered statistically significant.

Third, we examined the impact of LTCI on CHLTCE among subgroups with different health conditions (RQ2). As noted in the section ‘Impact of Long-term Care Insurance on reducing health and long-term care costs’, the effects of LTCI on CHLTCE may differ depending on the level of CI, physical limitations, and chronic diseases. We compared the coefficient (the impact of LTCI) for different subgroups to investigate the differential role of LTCI in CHLTCE based on the level of CI, physical limitations, and chronic diseases.

We conducted two sets of robustness checks. First, we applied an alternative cutoff on the 0–11 scale: following prior studies (Lee et al. 2018; Lin et al. 2025), individuals with a cognitive summary score less than 1.5 standard deviations below the population mean—stratified by education levels—were classified as having CI. The staggered DID regression analysis was then repeated. Second, the 10% threshold of total household expenditures was used as an additional criterion for catastrophic expenditures. Subsequently, staggered DID analyses were conducted.

## Results

Table 1 presents the weighted descriptive characteristics across four waves. Among Chinese participants, the likelihood of experiencing CHLTCE was 12.5% at the 20% threshold and 11.2% at the 25% threshold of total household expenditures, respectively. Besides, older adults in the treated group were on average more likely to be younger, male, educated, married or cohabiting compared to those in the control group.

**Fig. 1:**
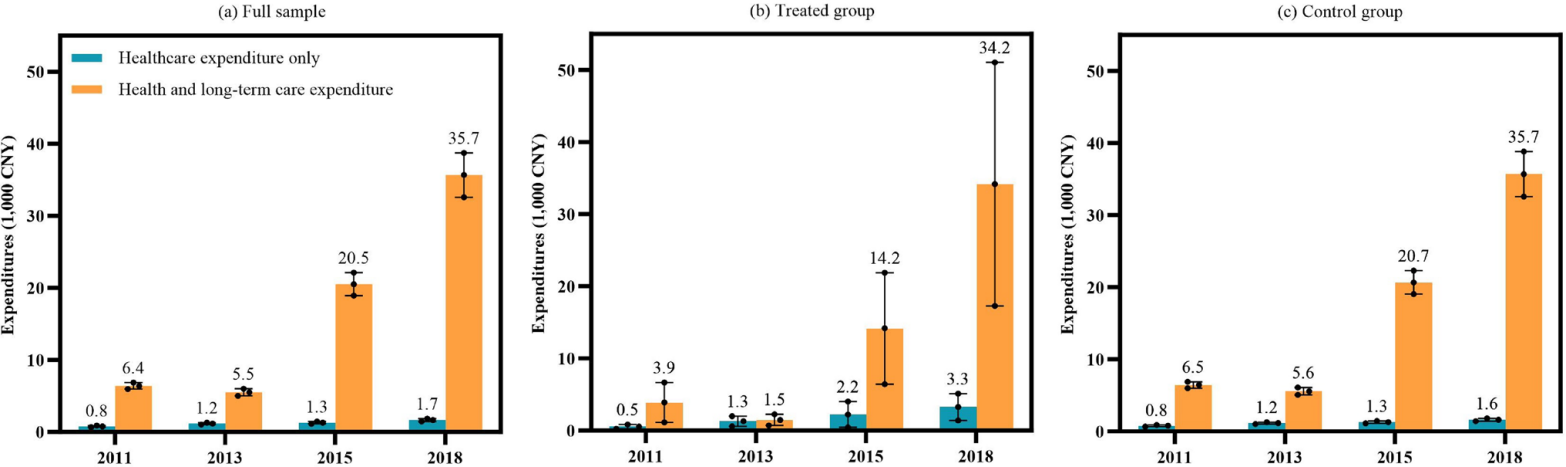
Weighted health and long-term care expenditures after accounting for long-term care costs. Longitudinal weight was applied

Figure 1 compares weighted health and LTC expenditures over time using two measures: one considering only healthcare expenditures and the other including both health and LTC expenditures. In the full sample (Fig. 1a), total health and LTC expenditures were consistently higher than when only healthcare costs were considered at each time point. A similar pattern was observed in both the treated group (covered by LTCI, Fig. 1b) and the control group (not covered by LTCI, Fig. 1c) in 2015 and 2020, where total expenditures were significantly higher than when only healthcare costs were considered.

**Fig. 2:**
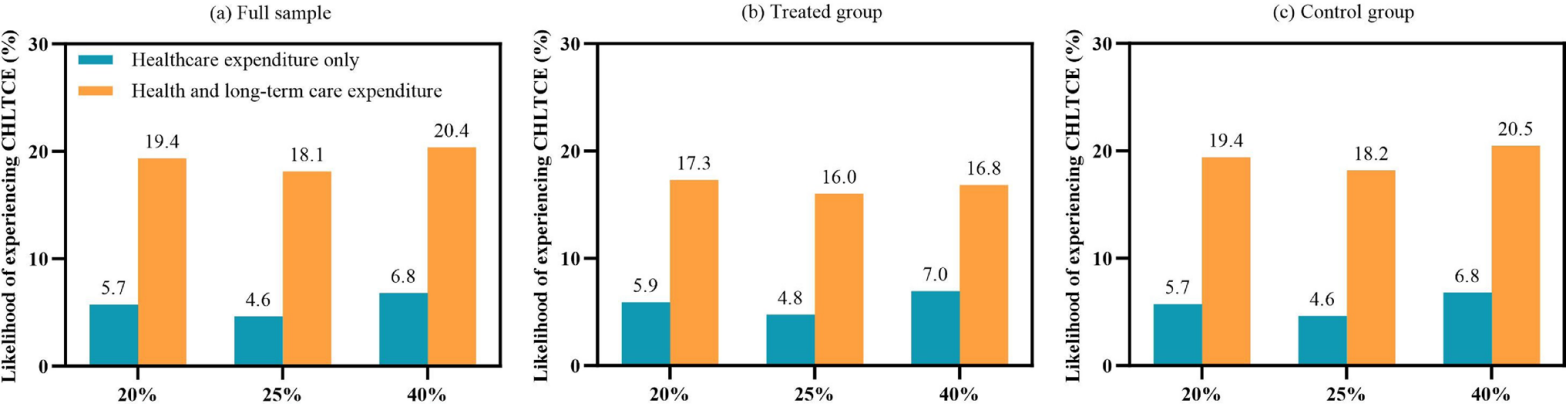
Weighted likelihood of experiencing catastrophic health and long-term care expenditure (CHLTCE) after accounting for long-term care costs. 20% and 25% refer to the 20% threshold and the 25% threshold of total household expenditures. 40% refers to the 40% threshold of non-food household expenditures. Longitudinal weight was applied

Figure 2 presents the weighted likelihood of experiencing CHLTCE after incorporating LTC costs. The results indicate an increase in the likelihood of experiencing CHLTCE across all groups. In addition, after accounting for LTC costs, the treated group (Fig. 2b) exhibited a lower likelihood of experiencing CHLTCE across all thresholds compared to the control group (Fig. 2c).

Table 2 presents the impact of LTCI on CHLTCE among the full sample. LTCI coverage significantly reduced the likelihood of experiencing CHLTCE by 10% and 9% at the 20% and 25% thresholds of total household expenditures, and by 18% at the 40% threshold of non-food household expenditures. Appendix Table H1 reports the full results.

**Table 2:** Effect of long-term care insurance on the likelihood of experiencing catastrophic healthcare and long-term care expenditure

	20% of total expenditures	25% of total expenditures	40% of non-food expenditures
Full sample	β ^P^ (robust S.E.)		
The net impact of long-term care insurance	− 0.10* (0.04)	− 0.09* (0.04)	− 0.18*** (0.04)
*Controls*			
demographic factors	Yes	Yes	Yes
socioeconomic factors	Yes	Yes	Yes
heath-related factors	Yes	Yes	Yes
Year fixed effects	Yes	Yes	Yes
Individuals fixed effects	Yes	Yes	Yes

20% and 25% refers to the 20% threshold and the 25% threshold of total household expenditures. 40% refers to the 40% threshold of non-food household expenditures. S.E. is the standard error. N = 31,076. *** *p* < 0.001, ** *p* < 0.01, * *p* < 0.05

**Fig. 3:**
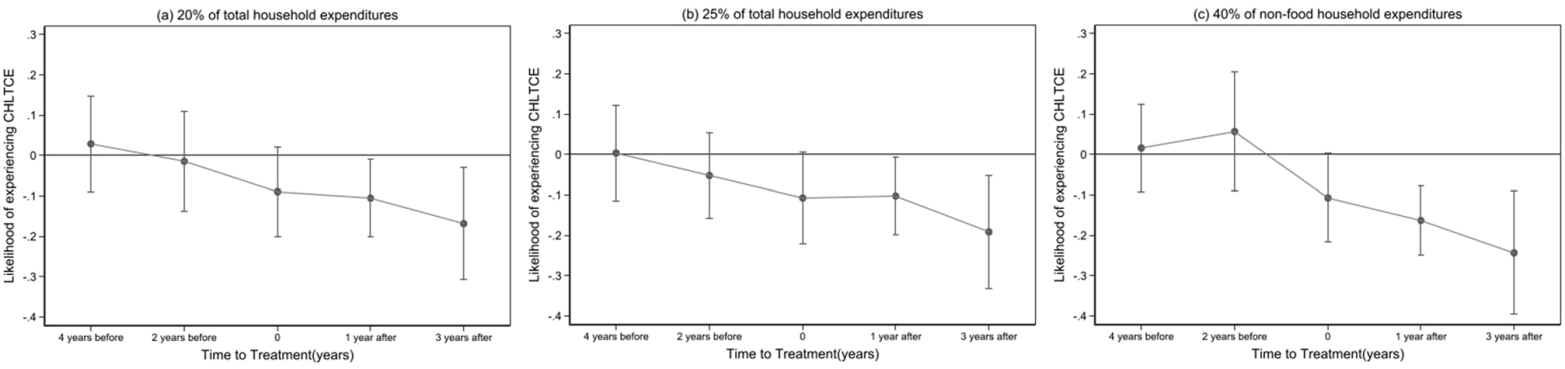
Event study for the effect of long-term care insurance on catastrophic health and long-term care expenditure (CHLTCE)

Figure 3 shows the results of the event study analysis. The estimates of the likelihood of experiencing CHLTCE for the pre-reform periods at all estimated thresholds were not significant, indicating that the pre-reform trends of CHLTCE in the treated group did not significantly differ from those in the control group. A significant decrease in the likelihood of experiencing CHLTCE was noted after the reform across all estimated thresholds.

Table 3 presents the results of subgroup analyses by cognitive function scores, physical limitations, and chronic diseases. For older adults with more severe CI (cognitive function score: 0–2), LTCI coverage reduced the likelihood of experiencing CHLTCE by 17% and 19% at the 20% and 25% thresholds of total household expenditures, respectively, and by 17% at the 40% threshold of non-food household expenditures. In contrast, among those with milder CI (cognitive function score: 3–5), no significant effect was observed at any threshold. The middle panel of Table 3 shows that for both groups—older adults with both cognitive and physical limitations and those with physical limitations but without CI—LTCI coverage only reduced the likelihood of experiencing CHLTCE at the 40% threshold of non-food household expenditures. However, it did not have a significant effect at the lower thresholds. Similarly, the lower panel of Table 3 shows that LTCI coverage reduced the likelihood of experiencing CHLTCE by 18% at the 40% threshold of on-food household expenditures among older adults with both physical limitations and chronic diseases. However, no significant effect was observed at any thresholds among older adults who had physical limitations but without chronic diseases. Supplement I presents the results of the robustness checks, which are consistent with the main findings.

**Table 3:** Subgroup analyses: effect of long-term care insurance on the likelihood of experiencing catastrophic health and long-term care expenditure (CHLTCE)

	20% of total expenditures	25% of total expenditures	40% of non-food expenditures
	*β*^P^ (robust S.E.)		
*Individuals with relatively severe CI only (cognitive function score* = *0–2)*			
The net impact of long-term care insurance	− 0.17* (0.08)	− 0.19* (0.09)	− 0.17* (0.08)
Observations	6228	6228	6228
*Individuals with milder levels of CI only (cognitive function score* = *3–5)*			
The net impact of long-term care insurance	− 0.06 (0.07)	-0.07 (0.07)	-0.09 (0.07)
Observations	3731	3731	3731
*Individuals with both cognitive and physical limitations*			
The net impact of long-term care insurance	− 0.26 (0.15)	− 0.25 (0.14)	− 0.41* (0.17)
Observations	8512	8512	8512
Individuals with physical limitations but without CI			
The net impact of long-term care insurance	− 0.08	− 0.06	− 0.13*
	(0.06)	(0.06)	(0.06)
Observations	18,267	18,267	18,267
*Individuals with physical limitations and chronic disease*			
The net impact of long-term care insurance	− 0.10 (0.05)	− 0.07 (0.06)	− 0.18** (0.06)
Observations	19,309	19,309	19,309
*Individuals with physical limitations but without chronic diseases*			
The net impact of long-term care insurance	0.02	0.02	0.12
	(0.21)	(0.21)	(0.28)
Observations	7470	7470	7470
Year fixed effects	Yes	Yes	Yes
Individuals fixed effects	Yes	Yes	Yes
Control variables	Yes	Yes	Yes

0% and 25% refers to the 20% threshold and the 25% threshold of total household expenditures. 40% refers to the 40% threshold of non-food household expenditures. S.E.: the standard error. CI: cognitive impairments. FE: fixed effects. *** *p* < 0.001, ** *p* < 0.01, * *p* < 0.05

## Discussion

Our study is one of the first to examine CHLTCE and the impact of LTCI on older adults with cognitive and/or physical limitations. We introduced the novel concept of CHLTCE by incorporating out-of-pocket LTC costs, formal care costs, and informal care costs to provide a better comprehensive assessment of how these limitations impact household living standards. This approach should be emphasized in future research, as previously overlooked LTC expenditures can significantly contribute to overall health and LTC costs for individuals with these conditions.

The results suggest that LTCI significantly reduces CHLTCE risks among older adults with cognitive and/or physical limitations. This finding aligns with previous studies indicating that China’s LTCI helps lower health and LTC costs (Feng et al. 2020; Lei et al. 2022). Nevertheless, prior research has not examined its impact on catastrophic expenditures. This study contributes to the literature by providing evidence of its role in alleviating such financial challenges. Moreover, the results indicate that the effects of LTCI on CHLTCE are consistent across the two groups: older adults with both cognitive and physical limitations, and those with physical limitations but no CI. We found no indication that LTCI has a stronger effect among those with both cognitive and physical limitations, despite their greater care needs and potential for higher expenditures. This may be because eligibility for LTCI is primarily determined by physical limitations rather than CI (Zhou & Yuan 2021). While all pilot cities provide coverage for individuals with moderate or severe physical impairments, only six cities extend coverage to those with CI (Hao and Li 2021; Zhou and Yuan 2021).

Moreover, the findings suggest that the impact of LTCI on CHLTCE varies depending on the severity of CI. It reduces CHLTCE risks across all thresholds for older adults with more severe CI but does not have the same effect for those with milder CI. Previous research suggests that China’s LTCI applies strict eligibility criteria (Zhou and Dai 2021). In most pilot cities, it does not directly cover individuals with mild CI (Hao and Li 2021). Instead, these individuals must meet additional criteria—such as having moderate or severe physical limitations and being enrolled in urban health financing schemes—to qualify for benefits (Hao and Li 2021). These restrictions exclude many older adults with mild CI, especially rural residents (Zhu and Oesterle 2019). Furthermore, LTCI provides higher reimbursement for LTC services to individuals assessed as having greater LTC needs (Zhou and Dai 2021). Evidence indicates that individuals with severe CI are more likely to experience dual functionality loss, encompassing both physical and cognitive limitations (Ferraro et al. 2023). Therefore, they are more likely to be classified as having critical LTC needs, making them eligible for higher LTCI benefits.

Furthermore, the provision of greater financial support to individuals with more severe LTC needs (Zhou and Dai 2021) may also explain why LTCI reduces CHLTCE risks at the 40% threshold among older adults with both physical limitations and chronic diseases, but not among those with only physical limitations and no chronic disease. Previous research has shown that chronic diseases are associated with higher risks of physical limitations (Kyu et al. 2016), making individuals with both physical limitations and chronic diseases more likely to be classified as having critical LTC needs and, therefore, eligible for LTCI benefits.

In addition, we found that among older adults with both cognitive and physical limitations, LTCI’s effect on reducing CHLTCE risks is observed only at the high threshold (40% of non-food expenditures) but not at lower thresholds (20% and 25% of total expenditures). This may be due to insufficient coverage and greater care needs. LTCI does not cover all care services, and the co-payment rates for out-of-pocket formal care expenditures after reimbursement typically range from 25 to 50% in most pilot cities (Pei et al. 2024). Evidence shows that out-of-pocket expenses exceeding insurance limits still pose a significant risk of catastrophic expenditures for beneficiaries (Liu et al. 2023). Similarly, a study in Spain found that, despite the existing public LTC coverage, risk mitigation remained insufficient, and catastrophic expenditures persisted (Guillen and Comas-Herrera 2012). Moreover, older adults with both cognitive and physical limitations often require more extensive care and incur higher lifetime expenditures than those without such limitations (Kwak et al. 2021). While LTCI may help alleviate CHLTCE risks at a high threshold, its impact appears insufficient when assessed at a lower threshold, where financial strain remains substantial even after insurance reimbursement.

Our findings have significant policy implications for the expansion and reform of LTCI programs in low- and middle-income countries. Firstly, in most pilot cities, current LTCI eligibility rules apply tiered coverage primarily based on physical limitations levels (Zhou and Yuan 2021). However, we found that LTCI is particularly effective in reducing CHLTCE risks among older adults with more severe CI and those with both physical limitations and chronic diseases. To strengthen this effect, policymakers should consider adjusting eligibility criteria and implementing tiered coverage based on broader aspects of cognitive and physical function—beyond just ADLs—to provide more targeted financial protection for individuals with more complex care needs. Secondly, our findings indicate that while LTCI offers financial protection for older adults with complex health conditions, its impact in reducing CHLTCE risks is primarily observed at higher expenditure thresholds. This suggests that current coverage may be insufficient for many beneficiaries. We suggest policymakers consider increasing reimbursement rates or reducing out-of-pocket costs to ensure that LTCI provides more comprehensive support, particularly for those who continue to face significant financial challenges despite receiving insurance benefits. Thirdly, we found that older adults with mild CI are less likely to be protected by LTCI, despite their vulnerability to CHLTCE. Expanding coverage to include this population could help mitigate their financial risks and improve overall access to necessary care services.

Our study has several limitations. Firstly, as out-of-pocket LTC expenditures are not recorded in the 2018 wave of CHARLS, LTC costs were estimated based on the formal and informal care hours received and the corresponding average national hourly payment (Van den Berg et al. 2006). This approach may introduce some bias. Secondly, ideally, we would have also liked to consider indirect healthcare expenditures (e.g., travel costs associated with receiving healthcare). However, our data do not support the collection of these costs at all waves of CHALRS. Thirdly, we did not include insurance premiums in the calculation of health and LTC costs because these premiums vary by city and survey year, and obtaining the corresponding data is challenging. Future research will incorporate these premiums. Fourth, the same questions on cognitive function were not asked at each wave of CHARLS. Therefore, we were unable to use the complete set of 30 Mini-Mental State Examination items to construct our indicator and instead drew on the 11 questions consistently asked in all waves similar to prior studies (Li et al. 2022). It cannot be considered a clinical measure of CI. Lastly, ideally, we would like to have a larger treatment group. However, the treatment group of older adults with cognitive and/or physical limitations consists of 558 observations. Although the sample size is relatively limited, it is comparable to previous studies using the DID approach to evaluate the impact of LTCI (Chen and Ning 2022; Lei et al. 2022). Due to data constraints, we were unable to expand the treated group. This limitation also influenced our decision not to focus exclusively on individuals with CI, as doing so would have resulted in a smaller sample size.

## Conclusion

Our findings indicate that estimating catastrophic expenditures among older adults with cognitive and/or physical limitations solely based on healthcare costs could underestimate their financial challenges. Besides, China’s LTCI can reduce CHLTCE risk in this population and offer greater financial protection for older adults with severe cognitive impairment and for those with both physical limitations and chronic diseases. Our results have significant policy implications for expanding LTCI initiatives in China, refining eligibility criteria, and advancing LTCI programs in other low- and middle-income nations facing similar challenges of population aging and growing LTC demands.

## Supplementary Information

Below is the link to the electronic supplementary material.

Supplementary file1 (DOCX 198 KB)

## Data Availability

The data that support our study are available from the China Health and Retirement Longitudinal Study, Peking University, Beijing, China (https://charls.pku.edu.cn/).
