# Supplementary material for: Can long-term care insurance reduce catastrophic health and long-term care expenditures among older adults? A quasi-experimental study in China
Source: Eur J Ageing. 2025 Jun 3;22(1):25. doi: 10.1007/s10433-025-00861-1 (PMC12134244; doi:10.1007/s10433-025-00861-1)
Supplement: Supplementary file 1 — Supplementary file1 (DOCX 198 KB) [file 10433_2025_861_MOESM1_ESM.docx]

**Supplementary Materials**

**Contents**

[Section A: Pilot cities and key features of long-term care insurance in China 1](#_Toc195209559)

[Table A.1. Key features of the long-term care insurance in China. 2](#_Toc195209560)

[Section B: Measurement of cognitive function and physical limitations 3](#_Toc195209561)

[Section C. Flow chart of the study sample 4](#_Toc195209562)

[Figure C.1. Flow chart of the study sample 4](#_Toc195209563)

[Section D: Measurement of dependent variables 5](#_Toc195209564)

[Section E. Treated group, control group and the timing of long-term care insurance pilot schemes 8](#_Toc195209565)

[Table E.1 The timing of policy implementation, population coverage, and copayment rates of 16 pilot cities in our study sample. 8](#_Toc195209566)

[Section F: Measurement of control variables 10](#_Toc195209567)

[Section G: Identification strategy 11](#_Toc195209568)

[Section H: Full results of the main models 13](#_Toc195209569)

[Table H.1: Effect of long-term care insurance on the likelihood of experiencing of catastrophic health and long-term care expenditure. 13](#_Toc195209570)

[Section I: Robustness check 16](#_Toc195209571)

[Figure I.1. Robustness check 1: Weighted likelihood of experiencing catastrophic health and long-term care expenditure (CHLTCE) after accounting for long-term care costs. 16](#_Toc195209572)

[Table I.1: Robustness check 1: Effect of long-term care insurance on the likelihood of experiencing of catastrophic health and long-term care expenditure. 16](#_Toc195209573)

[Figure I.2. Robustness check 2: Weighted likelihood of experiencing catastrophic health and long-term care expenditure (CHLTCE) after accounting for long-term care costs. 19](#_Toc195209574)

[Table I.2: Robustness check 2: Effect of long-term care insurance on the likelihood of experiencing of catastrophic health and long-term care expenditure. 19](#_Toc195209575)

# **Section A: Pilot cities and key features of long-term care insurance in China**

The Chinese government initiated the first round of long-term care insurance (LTCI) scheme in 15 cities and two provinces (Jilin and Shandong) in July 2016 (Lei et al., 2022). Among the 15 cities, only 12 were included in our study sample. Specifically, three pilot cities (Shihezi, Changchun, and Nantong) are not in our study sample. Among the two pilot provinces, four cities (Liaocheng, Weifang, Linqi, and the Jilin city) have also initiated the pilot phase of LTCI since 2017, and these four cities are included in our study sample. Therefore, there are 16 pilot cities in our study sample (Lei et al., 2022): Shanghai, Qingdao, Jingmen, Guangzhou, Liaocheng, Weifang, Linqi, Chengdu, Chengde, Jilin, Qiqihaer, Anqing, Ningbo, Chongqing, Shangrao, and Suzhou. Table S2 summarizes the key features of China’s long-term care insurance.

## Table A.1. Key features of the long-term care insurance in China.

| Fund pooling |  | 1) use Social Health Insurance funds solely (e.g, Ningbo)  2) two (e.g., Qiqihaer) or three (e.g., Chengde) grant schemes, including Social Health Insurance, financial assistance, employers’ contributions, personal payments, and welfare lottery funds |
| --- | --- | --- |
| Purchasing | Hospitals | 40-150 CNY per day for care provided. |
|  | Nursing home | 23-120 CNY per day for nursing homes. |
|  | Home-based care | 20-105 CNY per day for home-based care. |
| Services covered | Hospitals | For institutional care provided at hospitals and nursing homes, LTCI covers nursing care provided by designated caregivers. Patients are responsible for payments related to food, travel, accommodation, and carer costs. |
|  | Nursing home |  |
|  | Home-based care | Regular visits from home care assistants (depending on the pilot cities). |
| Eligibility | Eligible participants need to meet the following criteria (varying by pilot cities):   1. have undergone a long-term care needs assessment and have been determined to have certain levels of needs 2. be residents enrolled in UEI, URI, or URMI (depending on the pilot cities) | |
| Premiums and copayments | An annual contribution of RMB 20-180 for UEI a RMB 10-90 for UEI or URRMI. Copayments can be found in Table S2 | |

*Notes*. UEI: Urban Employee Insurance. URI: Urban Resident Employee Insurance. URRMI: Urban-Rural Resident Medical Insurance. Source: (National Healthcare Security Administration of China & Ministry of Finance of China, 2020; Pei et al., 2024; Yang et al., 2018; Zhou & Dai, 2021)

# **Section B: Measurement of cognitive function and physical limitations**

**Measure of cognitive function**

Since the questions measuring cognitive function at each wave of the China Health and Retirement Longitudinal Survey (CHARLS) are inconsistent, we followed the measure used in previous research (Li et al., 2022) and calculated cognitive function by summing the number of correct responses to the 11 questions consistently asked in all waves. The total score ranges from 0 to 11, with a higher score indicating better cognitive function. Questions asked included today’s date (month, day, year, and seasons, four points), the day of the week (one point), the serial subtraction of seven from 100 (five times, five points), and figure drawing (one point) (Li et al., 2022). In figure drawing, participants were asked to redraw a picture from a picture (one point) (Li et al., 2022). Consistent with previous studies (Jak et al., 2009; Li et al., 2022), the respondent was categorized as having cognitive limitations if the summary score was less than six.

**Measure of physical limitations**

Physical limitations were measured by older adults’ ability to perform daily tasks (Hu et al., 2022). Participants were asked to report whether or not they had difficulties in performing six activities of daily living (ADLs; dressing, bathing, eating, walking across a room, using the toilet, and controlling urination), five instrumental activities of daily living (IADLs; doing household chores, cooking, shopping, taking medications, and managing money), and six mobility tasks (walking 100m, climbing several flights of stairs, getting up from a chair, stooping or kneeling or crouching, extending arms up, lifting 5kg, and picking up a small coin). We followed previous research (Hu et al., 2022) and created a dichotomous variable to identify older adults with physical limitations: 1 = have at least one difficulty with any ADLs, IADLs, or mobility tasks; 0 = none difficulties.

# **Section C. Flow chart of the study sample**

## **Figure C.1. Flow chart of the study sample**

**
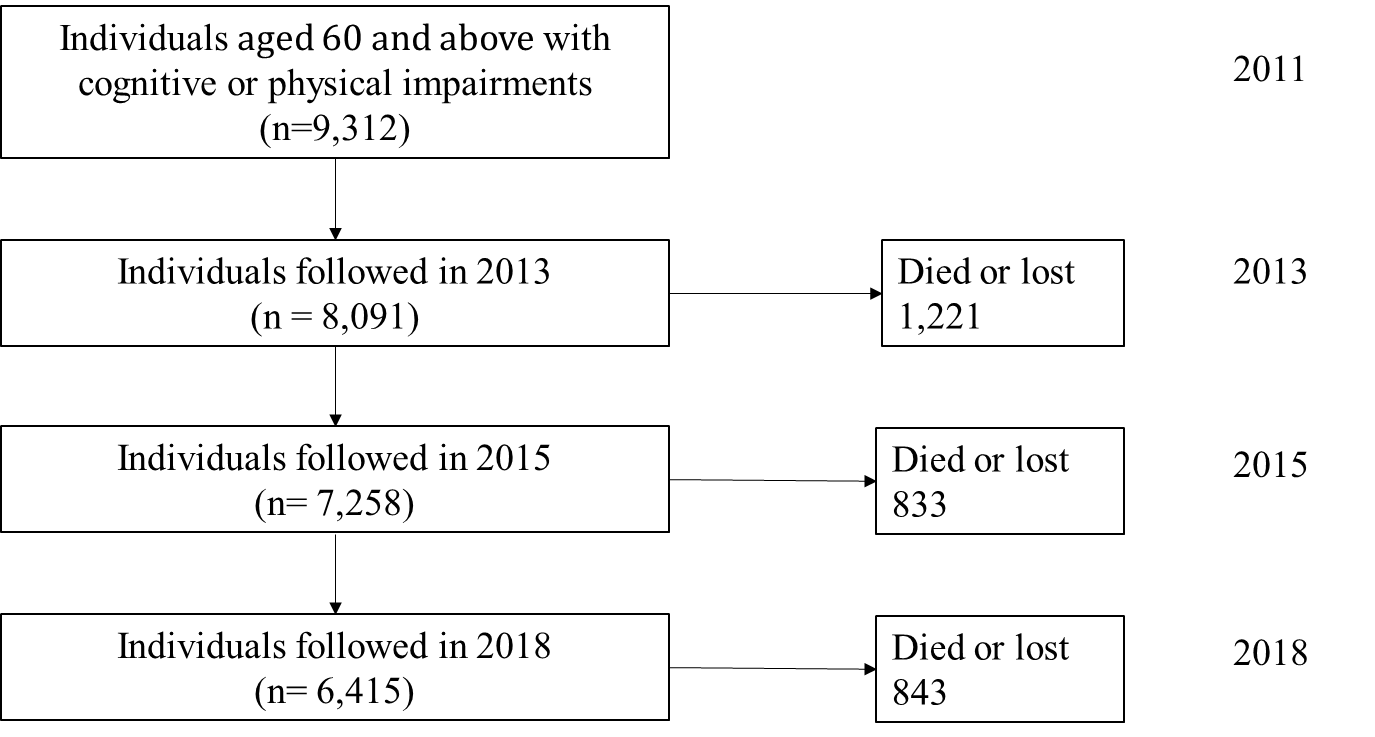
**

# **Section D: Measurement of dependent variables**

The outcome in our study is the likelihood of experiencing catastrophic health and long-term care expenditures (CHLTCE). These indicators were calculated using measures of total health and long-term care (LTC) costs by aggregating out-of-pocket healthcare expenditures, out-of-pocket formal care costs, and informal care costs. In terms of healthcare expenditures, respondents in CHARLS were asked to report out-of-pocket expenditures in the previous year (e.g., inpatient and outpatient expenditures) (Yang & Hu, 2022). We did not include indirect healthcare expenditures here (e.g., travel costs associated with receiving healthcare) as our data do not support the collection of these costs at all waves of CHALRS.

In terms of LTC costs, since out-of-pocket LTC expenditures were not recorded in the CHARLS, these costs were estimated by calculating the value of both formal and informal care individuals received. The calculation of out-of-pocket formal LTC costs followed these four steps. Firstly, following previous studies (Hu et al., 2022), individuals with LTC needs as those having at least one difficulty in performing any ADLs, IADLs, or mobility tasks. Secondly, LTC was classified as ‘informal’ when the carer was a family member or an unpaid individual without any affiliation to an institution (Hurd et al., 2013). Other forms of care, whether acquired through institutional care, community-based care, or hired helpers, were categorized as ‘formal’ LTC was classified as "formal" which was acquired through institutional care, community-based care, or hired helpers (Hurd et al., 2013). We identified individuals with LTC needs who received formal care and calculated the total hours of formal care they received in the previous month. As in previous research (Zhao et al., 2020), the monthly formal hours of LTC were multiplied by 12 to establish a yearly indicator for formal care hours received. Thirdly, we estimated the costs for formal care by multiplying the formal care hours received by the average national hourly payment for institutional care (China National Bureau of Statistics, 2019).

Fourthly, considering that the LTCI covers formal care costs for eligible individuals, we examined the copayment rates for formal LTC expenses for individuals in each LTCI pilot city who are eligible for LTCI (see Supplementary Table S1). Specifically, we estimated their out-of-pocket costs for formal LTC by multiplying their annual formal care costs by the corresponding copayment rate. For instance, in Shanghai, the out-of-pocket for formal care costs (1,500 CNY) are derived by applying a copayment rate of 15% to the individual's annual formal care costs (10,000 CNY). In some pilot cities, the copayment rate for institutional care expenses is not applicable (Table S1). To estimate the out-of-pocket costs for formal LTC in these cases, we subtracted the annual reimbursement payments for LTCI beneficiaries from the estimated total formal LTC costs in the preceding year. For instance, in Ningbo in 2018, we deducted the hourly reimbursement payments for LTCI beneficiaries (40 CNY/hour) from the average national hourly payment for private care (49 CNY/hour) (China National Bureau of Statistics, 2019). Subsequently, we estimated the out-of-pocket formal care costs (6,480 CNY), which were calculated by multiplying the payment difference (9 CNY) by the total hours of formal LTC the individual had obtained in the previous year (60 hours/month * 12 months).

In addition, following the approach used in prior studies (Engel et al., 2021; Van den Berg et al., 2004), informal care costs were measured by valuing the time spent by informal carers based on forgone wages. The total hours of informal care received by individuals in the preceding month were calculated. Like the formal care measure, monthly hours of informal care were multiplied by 12 to create a yearly indicator for informal care hours received (Zhao et al., 2020). The monetary value of time spent by informal carers was then calculated as a forgone wage based on the average national hourly wage in the nursing or care sector (Equation 1), creating a continuous variable for informal care costs (Van den Berg et al., 2006).

Value informal care = t_i_w (1)

t_i_ is the time spent on ADL/IADL tasks by the informal carer i. w is the average national hourly wage (China National Bureau of Statistics, 2019).

We calculated the proportion of individuals’ health and LTC costs by aggregating out-of-pocket healthcare expenditures, out-of-pocket formal care costs, and informal care costs. The proportion of an individual’s health and LTC costs relative to their annual household expenditures was then calculated. Both measures were adjusted for inflation, expressed in 2011 prices using the Consumer Price Index (World Bank, 2022), and trimmed at the 1% quantile on both ends (Leone et al., 2019). According to World Health Organization and previous research (Yang and Hu, 2022, World Health Organization, 2024), different thresholds—20% and 25% of total household expenditures, as well as 40% of non-food household expenditures were used to determine CHLTCE in each survey wave. The 10% of total household expenditure threshold was not used, as previous research suggests that a low threshold would overestimate catastrophic expenditures (Arsenijevic et al., 2013; Wagstaff & van Doorslaer, 2003), especially for older adults with cognitive and/or physical limitations, who often face excessive health and LTC expenditures. An individual is marked as incurring CHLTCE if $\frac{H_{i}}{E_{i}}>z_{l}$, where ‘$H_{i}$’ is health and LTC costs of the individual i and ‘$E_{i}$’ refers to the household expenditures (World Health Organization, 2024). ‘$Z_{l}$’ is the predetermined CHLTCE threshold, defined as (210% of total household expenditures, 250% of total household expenditures, or 25% of non-food household expenditures. Specifically, we created three sets of dichotomous variables indicating whether the proportion of out-of-pocket health and LTC costs exceeded the defined threshold in each survey wave: a) individuals with CHLTCE at 20% (0 = out-of-pocket health and LTC costs < 20% of total household expenditures; 1= ≥ 20%); b) individuals with CHLTCE at 25% (0 = out-of-pocket health and LTC costs < 25% of total household expenditures; 1= ≥ 25%); c) individuals with CHLTCE at 40% (0 = out-of-pocket health and LTC costs < 40% of non-food household expenditures; 1= ≥ 40%).

# **Section E. Treated group, control group and the timing of long-term care insurance pilot schemes**

As noted in Section A and shown in **Table E.1**, our study sample includes 16 pilot cities (Lei et al., 2022). Following the approach used in previous studies (Lei et al., 2022; Pei et al., 2024), we defined the treatment group as individuals residing in these 16 pilot cities who were eligible for LTCI, and the control group as those residing in non-pilot cities (Pei et al., 2024). Specifically, individuals in the treatment group were required to meet both the following two criteria: (1) residing in a pilot city; and (2) being covered by an urban Social Health Insurance (SHI) scheme (e.g., Urban Employee Insurance). The specific SHI schemes linked to LTCI eligibility vary by the pilot city and are detailed in the third column of **Table E.1**. In addition, some pilot cities restricted LTCI eligibility to individuals aged 60 years and older. However, we did not list this age criterion in **Table E.1**, as our study sample includes only older adults (aged 60 and above) with cognitive and/or physical limitations. In contrast, the control group comprises individuals who resided in non-pilot cities across all study waves, regardless of their SHI coverage status (Pei et al., 2024).

## **Table E.1 The timing of policy implementation, population coverage, and copayment rates of 16 pilot cities in our study sample.**

| **Pilot cities** | **Implementation timing** | **Eligibility/Population coverage** | **Copayment rate** |
| --- | --- | --- | --- |
| Shanghai | 2017-1 | Enrolled in the UEI or URRMI | 15% |
| Qingdao | 2012-7 | Enrolled in the UEI or URRMI | 10% for UEI enrollees and 20%-30% for URRMI enrollees. |
| Jingmen | 2017-1 | Enrolled in the UMI or URRMI | 25% |
| Guangzhou | 2017-8 | Enrolled in the UEI | 25% |
| Liaocheng | 2017-10 | Enrolled in the UEI | 25% |
| Weifang | 2017-4 | Enrolled in the UEI | 25% |
| Linqi | 2017-8 | Enrolled in the UEI | 25% |
| Chengdu | 2017-6 | Enrolled in the UEI | 30% |
| Chengde | 2017-7 | Enrolled in the UEI | 30% |
| Jilin | 2016-12 | Enrolled in the UEI or URRMI | 30% |
| Qiqihaer | 2017-10 | Enrolled in the UEI | 45% |
| Anqing | 2017-1 | Enrolled in the UEI | 50% |
| Ningbo | 2017-12 | Enrolled in the UEI | N/A. Formal care recipients would be reimbursed 40 CNY per day*. |
| Chongqing | 2017-12 | Enrolled in the UEI | N/A. Formal care recipients would be reimbursed 50 CNY per day*. |
| Shangrao | 2017-9 | Enrolled in the UEI | N/A. Formal care recipients would be reimbursed 1200 CNY per month*. |
| Suzhou | 2017-6 | Enrolled in the UEI or URRMI | N/A. Formal care recipients would be reimbursed 26 CNY per day for those with severe disabilities and 20 CNY per day for those with moderate disabilities*. |

*Notes*: Sources: (National Healthcare Security Administration of China & Ministry of Finance of China, 2020; Pei et al., 2024; Yang et al., 2018; Zhou & Dai, 2021). UEI is Urban Employee Insurance, which covers urban employees in formal sectors. URRMI is Urban-Rural Resident Medical Insurance, which covers rural residents and urban residents without employment in the formal sectors, including the self-employed, unemployed individuals, retirees, young children, and primary/secondary school students. The copayment rate here refers to the proportion of out-of-pocket formal LTC expenditures, after government reimbursement by LTCI, relative to the total LTC expenditures.

# **Section F: Measurement of control variables**

Age is a continuous variable measured by years. Gender is a binary variable with ‘male’ set as the reference category. Education is categorized as follows: ‘no formal education’ (the reference group), ‘elementary school’, and ‘middle school or above’. Marital status has two categories: ‘married/cohabiting’ (the reference group) and ‘other’ (including being widowed, separated, divorced, or never married). Place of residence is a binary variable, distinguishing between ‘rural’ and ‘urban’ areas (with ‘urban’ as the reference group). Annual household expenditure was adjusted for inflation, expressed in 2011 prices using the Consumer Price Index (World Bank, 2022), and trimmed at the 1% quantile on both ends (Leone et al., 2019). Social Health Insurance (SHI) status is categorized as follows: ‘Urban Employee Basic Medical Insurance (UEBMI)’; ‘Urban Resident Basic Medical Insurance (URBMI)’, ‘New Rural Cooperative Medical Scheme (NCMS)’, ‘Urban and Rural Resident Basic Medical Insurance (URRMI), and ‘other’. Self-rated health has three categories: ‘good’ (the reference group), ‘fair’, and ‘bad’ status. The CHARLS questionnaire asked survey participants whether they need others' help or have difficulties performing six ADLs (eating, dressing, bathing, using the toilet, continence, and getting in and out of bed) and five IADLs (cooking, shopping, making phone calls, taking medication, and doing housework). We summed up the total number of reported ADL limitations and IADL limitations among participants, ranging from 0 to 11, with a higher score suggesting greater limitations. Depressive symptoms were assessed using the 10-item Centre for Epidemiologic Studies—Depression (CES-D) scale (Yang & Hu, 2022). Participants were required to evaluate eight negative statements (e.g., I felt fearful) and two positive statements (e.g., I felt hopeful) to reflect their mental health status over the past week. Each statement was rated on a 4-point scale: from 1 (less than one day), 2 (one to two days), 3 (three to four days), and 4 (five to seven days) (Yang & Hu, 2022). The two positive statements were reverse-scored; then, the scores of all 10 statements were aggregated. This resulted in a score capturing depressive symptoms ranging from 10 (no symptoms) to 40 (severe symptoms) (Yang & Hu, 2022).

# **Section G: Identification strategy**

The analyses in our study consist of three steps. First, we compared health-related expenditures and the likelihood of experiencing CHLTCE using two measures: one solely considering out-of-pocket healthcare expenditures and the other also including formal and informal LTC costs. Individual longitudinal weights with household and individual response adjustments were applied to the summary statistics.

Second, to answer the first research question (RQ1), we conducted staggered difference-in-differences (DID) analyses with individual [fixed effects](https://www.sciencedirect.com/topics/social-sciences/fixed-effects-model) to analyze the impact of LTCI on CHLTCE. The settings were DID with staggered adoption because the timing of policy implementation varied across the pilots (Pei et al., 2024). As noted in the independent variable section, enrollers of the LTCI in pilot cities are included in the treatment group and the control group consists of the observations in non-pilot cities. We adopted the staggered DID developed by Sun and Abraham (2021) as follows:

y_ct_ = α_i_ + λ_t_ +∑_g∉C_ ∑_d≠-1_ β_g,d_(1{E_i_=g}*$D_{it}^{d}$) + ε_it_

where y_ct_ denotes the outcome variable: the likelihood of experiencing CHLTCE, for individual _i_ at time _t_. E_i_ is the time when individual _i_ is initially covered by the LTCI, which is equal to “∞” for never-treated individuals. Whether an individual is covered by the LTCI or not can be determined by their eligibility rule (refer to Independent variable section for detailed information). g ∈{2012, 2015, 2017, ∞}, indicating disjoint cohorts. We set C = {∞} because there is a never-treated cohort. d denotes the relative time between E_i_ and _t_. $D_{it}^{d}$ is an indicator for individual i being d periods away from the initial treatment at time _t_. For the treatment groups, $D_{it}^{d}$=1 and individual i is in d periods away from the initial treatment at time _t_. For never-treated individuals, $D_{it}^{d}$=0. β is the estimation of the net impact of expanding LTCI coverage. α controls for the individual effects and λ is the year fixed effects. ε_ct_ is a random error term. Standard errors are clustered at the individual level to account for possible correlations in outcomes between individuals. Our results show that the potential trends in the outcome variables in both the treated and control groups were parallel without the implementation of LTCI. P-values < 0.05 were considered statistically significant.

Third, we examined the impact of LTCI on CHLTCE among subgroups with different health conditions (RQ2). As noted in the section ‘Impact of Long-term Care Insurance on reducing health and long-term care costs’, LTCI offers different benefits for LTC services based on the cognitive and physical conditions of beneficiaries. Consequently, the relationship between LTCI and CHLTCE may differ depending on the level of CI, physical limitations, and chronic diseases. We compared the coefficient (the impact of LTCI) for these subgroups to investigate the differential role of LTCI in CHLTCE based on the level of CI, physical limitations, and chronic diseases.

We conducted two sets of robustness checks. First, we applied an alternative cutoff along the 0-11 scale to assess whether the findings varied across different definitions of cognitive impairments. Following prior studies (Lee et al., 2018; Lin et al., 2025), individuals with a cognition summary score less than 1.5 standard deviations below the population mean, stratified by education levels, were classified as having cognitive impairment. The staggered Differences-in-Difference regression analysis was then repeated. Second, the 10% threshold of total household expenditures as an additional criterion for catastrophic expenditures. Subsequently, staggered DID analyses were conducted.

# **Section H: Full results of the main models**

## Table H.1: Effect of long-term care insurance on the likelihood of experiencing of catastrophic health and long-term care expenditure.

|  | **20% of total expenditures** | **25% of total expenditures** | **40% of non-food expenditures** |
| --- | --- | --- | --- |
| **Variables** | **β ^P^ (robust S.E.)** | |  |
| The net impact of long-term care insurance | -0.10*  (0.04) | -0.09*  (0.04) | -0.18***  (0.04) |
| Age (years) | 0.02*** | 0.02*** | 0.02*** |
|  | (<0.01) | (<0.01) | (<0.01) |
| Gender (Ref: male) |  |  |  |
| female | N.A. | N.A. | N.A. |
| Education (Ref: no formal education) | |  |  |
| elementary school | N.A. | N.A. | N.A. |
| middle school or above | N.A. | N.A. | N.A. |
| Place of residence (Ref: urban areas) | |  |  |
| rural areas | N.A. | N.A. | N.A. |
|  | N.A. | N.A. | N.A. |
| Household expenditures | -0.01** | -0.01*** | -0.01* |
|  | (<0.01) | (<0.01) | (<0.01) |
| Marital status (Ref: married or cohabiting) | | |  |
| other | 0.02 | 0.02 | 0.02 |
|  | (0.02) | (0.02) | (0.02) |
| Self-rated health (Ref: good) | |  |  |
| fair | 0.01 | 0.01 | 0.01 |
|  | (0.01) | (0.01) | (0.01) |
| bad | 0.05** | 0.04** | 0.05** |
|  | (0.02) | (0.02) | (0.02) |
| Number of ADL/IADL limitations | 0.12** | 0.12*** | 0.12*** |
|  | (0.01) | (0.01) | (0.01) |
| Type of Social Health Insurance | |  |  |
| UEI | 0.04 | 0.03 | 0.04 |
|  | (0.03) | (0.03) | (0.03) |
| URI | -0.01 | -0.03 | -0.03 |
|  | (0.03) | (0.03) | (0.04) |
| NCMS | 0.04 | 0.03 | 0.05* |
|  | (0.02) | (0.02) | (0.02) |
| URRMI | 0.02 | 0.01 | 0.03 |
|  | (0.03) | (0.03) | (0.04) |
| other | 0.02 | -0.01 | 0.01 |
|  | (0.03) | (0.03) | (0.03) |
| Depressive symptoms score | <0.01*** | <0.01*** | <0.01*** |
|  | (<0.01) | (<0.01) | (<0.01) |
| Year fixed effects | |  |  |
| 2013 | -0.09*** | -0.09*** | -0.09*** |
|  | (0.01) | (0.01) | (0.01) |
| 2015 | 0.02* | 0.01 | 0.03* |
|  | (0.01) | (0.01) | (0.01) |
| 2018 | N.A. | N.A. | N.A. |
| Constant | -1.18*** | -1.14*** | -1.53*** |
|  | (0.11) | (0.11) | (0.12) |
| Individuals fixed effects | Yes | Yes | Yes |

Notes: 20% and 25% refers to the 20% threshold and the 25% threshold of total household expenditures. 40% refers to the 40% threshold of non-food household expenditures. S.E. is the standard error. Other marital status refers to being separated, widowed, divorced, or never married. ADL is activities of daily living. IADL is instrumental activities of daily living. UEI is Urban Employee Insurance. URI is Urban Resident Insurance. NCMS is New Rural Cooperative Medical Scheme. URRMI is Urban-Rural Resident Medical Insurance. CI is cognitive impairments. FE is fixed effects. N.A. means not applicable. N=31,076. *** p<0.001, ** p<0.01, * p<0.05.

# **Section I: Robustness check**

**Figure I.1 and Table I.1** show the results of the first set of robustness checks, where cognitive impairment is defined as having a cognition summary score less than 1.5 standard deviations below the mean of the population, stratified by education levels (Lee et al., 2018; Lin et al., 2025). The findings in **Figure I.1** are consistent with the main results: (1) incorporating LTC costs led to an increased likelihood of experiencing CHLTCE across all groups; and (2) after accounting for these costs, the treated group exhibited a lower likelihood of experiencing catastrophic expenditures across all thresholds compared to the control group.

## Figure I.1. Robustness check 1: Weighted likelihood of experiencing catastrophic health and long-term care expenditure (CHLTCE) after accounting for long-term care costs.

Then, the staggered Differences-in-Differences regression analysis was repeated, as shown in **Table I.1**. The results aligned with the main models: LTCI coverage was associated with a significant reduction in the likelihood of experiencing CHLTCE across all estimated thresholds. This suggests that our findings remain robust when applying an alternative definition of cognitive impairments.

## Table I.1: Robustness check 1: Effect of long-term care insurance on the likelihood of experiencing of catastrophic health and long-term care expenditure.

|  | **20% of total expenditures** | **25% of total expenditures** | **40% of non-food expenditures** |
| --- | --- | --- | --- |
| **Variables** | **β ^P^ (robust S.E.)** | |  |
| The net impact of long-term care insurance | -0.10*  (0.04) | -0.09*  (0.04) | -0.18***  (0.04) |
| Age (years) | 0.02*** | 0.02*** | 0.02*** |
|  | (<0.01) | (<0.01) | (<0.01) |
| Gender (Ref: male) |  |  |  |
| female | N.A. | N.A. | N.A. |
| Education (Ref: no formal education) | |  |  |
| elementary school | N.A. | N.A. | N.A. |
| middle school or above | N.A. | N.A. | N.A. |
| Place of residence (Ref: urban areas) | |  |  |
| rural areas | N.A. | N.A. | N.A. |
|  | N.A. | N.A. | N.A. |
| Household expenditures | -0.01** | -0.01*** | -0.01 |
|  | (<0.01) | (<0.01) | (<0.01) |
| Marital status (Ref: married or cohabiting) | | |  |
| other | 0.01 | 0.01 | 0.01 |
|  | (0.02) | (0.02) | (0.03) |
| Self-rated health (Ref: good) | |  |  |
| fair | 0.01 | 0.01 | 0.02 |
|  | (0.01) | (0.01) | (0.01) |
| bad | 0.05** | 0.05** | 0.06** |
|  | (0.02) | (0.02) | (0.02) |
| Number of ADL/IADL limitations | 0.12*** | 0.12*** | 0.12*** |
|  | (0.01) | (0.01) | (0.01) |
| Type of Social Health Insurance | |  |  |
| UEI | 0.04 | 0.02 | 0.03 |
|  | (0.03) | (0.03) | (0.03) |
| URI | -0.02 | -0.04 | -0.04 |
|  | (0.03) | (0.03) | (0.04) |
| NCMS | 0.04 | 0.03 | 0.05* |
|  | (0.02) | (0.02) | (0.02) |
| URRMI | 0.02 | 0.00 | 0.02 |
|  | (0.03) | (0.03) | (0.04) |
| other | 0.01 | -0.01 | 0.00 |
|  | (0.03) | (0.03) | (0.04) |
| Depressive symptoms score | 0.00*** | 0.00*** | 0.00*** |
|  | (0.00) | (0.00) | (0.00) |
| Year fixed effects | |  |  |
| 2013 | -0.09*** | -0.09*** | -0.09*** |
|  | (0.01) | (0.01) | (0.01) |
| 2015 | 0.02* | 0.01 | 0.02 |
|  | (0.01) | (0.01) | (0.01) |
| 2018 | N.A. | N.A. | N.A. |
| Constant | -1.16*** | -1.12*** | -1.54*** |
|  | (0.12) | (0.12) | (0.13) |
| Individuals fixed effects | Yes | Yes | Yes |

Notes: 20% and 25% refers to the 20% threshold and the 25% threshold of total household expenditures. 40% refers to the 40% threshold of non-food household expenditures. S.E. is the standard error. Other marital status refers to being separated, widowed, divorced, or never married. ADL is activities of daily living. IADL is instrumental activities of daily living. UEI is Urban Employee Insurance. URI is Urban Resident Insurance. NCMS is New Rural Cooperative Medical Scheme. URRMI is Urban-Rural Resident Medical Insurance. N.A. means not applicable. N=31,076. *** p<0.001, ** p<0.01, * p<0.05.

**Figure I.2 and Table I.2** present the results of the second set of robustness checks, where a 10% threshold of total household expenditures was used as an additional criterion for catastrophic expenditures. The findings in **Figure I.2** align with the main results: (1) the likelihood of experiencing CHLTCE increased across all groups when LTC costs were included; and (2) after including LTC costs, the treated group had a lower likelihood of experiencing catastrophic expenditures compared to the control group.

## Figure I.2. Robustness check 2: Weighted likelihood of experiencing catastrophic health and long-term care expenditure (CHLTCE) after accounting for long-term care costs.


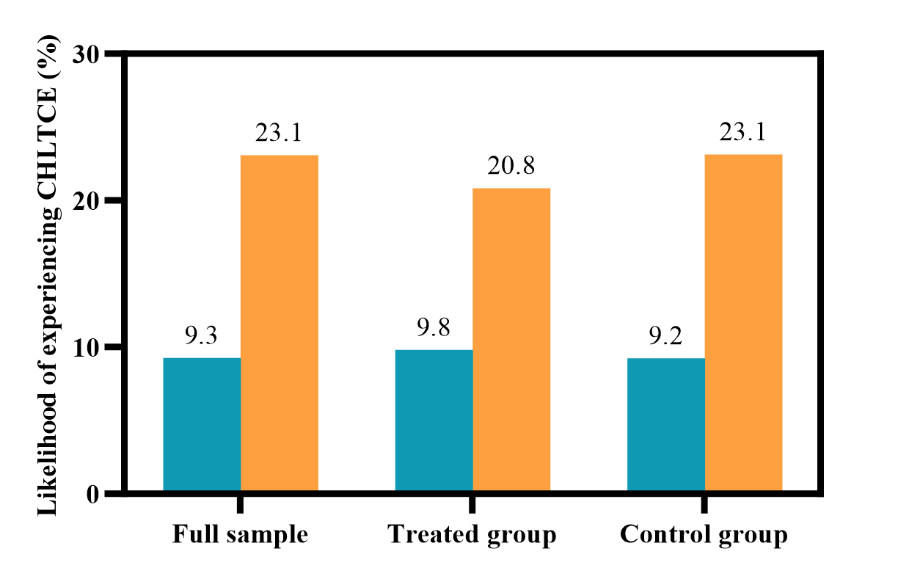


In addition, the results of staggered Differences-in-Differences regression (**Table I.2**) were consistent with the main models, showing that LTCI coverage was associated with a significant reduction in the likelihood of experiencing CHLTCE.

## Table I.2: Robustness check 2: Effect of long-term care insurance on the likelihood of experiencing of catastrophic health and long-term care expenditure.

|  | **10% of total expenditures** |
| --- | --- |
| **Variables** | **β ^P^ (robust S.E.)** |
| The net impact of long-term care insurance | -0.17***  (0.04) |
| Age (years) | 0.02*** |
|  | (<0.01) |
| Gender (Ref: male) |  |
| female | N.A. |
| Education (Ref: no formal education) | |
| elementary school | N.A. |
| middle school or above | N.A. |
| Place of residence (Ref: urban areas) | |
| rural areas | N.A. |
|  | N.A. |
| Household expenditures | -0.01* |
|  | (<0.01) |
| Marital status (Ref: married or cohabiting) |  |
| other | 0.05* |
|  | (0.02) |
| Self-rated health (Ref: good) | |
| fair | 0.02 |
|  | (0.01) |
| bad | 0.08*** |
|  | (0.02) |
| Number of ADL/IADL limitations | 0.12*** |
|  | (0.01) |
| Type of Social Health Insurance | |
| UEI | 0.04 |
|  | (0.03) |
| URI | -0.02 |
|  | (0.03) |
| NCMS | 0.03 |
|  | (0.02) |
| URRMI | 0.03 |
|  | (0.03) |
| other | <0.01 |
|  | (0.03) |
| Depressive symptoms score | <0.01*** |
|  | (<0.01) |
| Year fixed effects | |
| 2013 | -0.08*** |
|  | (0.01) |
| 2015 | 0.02 |
|  | (0.01) |
| 2018 | N.A. |
| Constant | -1.22*** |
|  | (0.12) |
| Individuals fixed effects | Yes |

Notes: 20% and 25% refers to the 20% threshold and the 25% threshold of total household expenditures. 40% refers to the 40% threshold of non-food household expenditures. S.E. is the standard error. Other marital status refers to being separated, widowed, divorced, or never married. ADL is activities of daily living. IADL is instrumental activities of daily living. UEI is Urban Employee Insurance. URI is Urban Resident Insurance. NCMS is New Rural Cooperative Medical Scheme. URRMI is Urban-Rural Resident Medical Insurance. N.A. means not applicable. N=31,076. *** p<0.001, ** p<0.01, * p<0.05.

**References**

Arsenijevic, J., Pavlova, M., & Groot, W. (2013). Measuring the catastrophic and impoverishing effect of household health care spending in Serbia. *Social Science & Medicine*, *78*, 17-25. https://doi.org/10.1016/j.socscimed.2012.11.014

China National Bureau of Statistics. (2019). *China Statistical Yearbook 2022*. http://www.stats.gov.cn/sj/ndsj/2022/indexch.htm

Engel, L., Ajdukovic, M., Bucholc, J., & McCaffrey, N. (2021). Valuation of informal care provided to people living with dementia: A systematic literature review. *Value in Health*, *24*(12), 1863-1870. https://doi.org/10.1016/j.jval.2021.04.1283

Hu, B., Cartagena-Farias, J., & Brimblecombe, N. (2022). Functional disability and utilisation of long-term care in the older population in England: A dual trajectory analysis. *European Journal of Ageing*, *19*(4), 1363-1373. https://doi.org/10.1007/s10433-022-00723-0

Hurd, M. D., Martorell, P., Delavande, A., Mullen, K. J., & Langa, K. M. (2013). Monetary costs of dementia in the United States. *New England Journal of Medicine*, *369*(5), 489-490. https://doi.org/10.1056/NEJMsa1204629

Jak, A. J., Bondi, M. W., Delano-Wood, L., Wierenga, C., Corey-Bloom, J., Salmon, D. P., & Delis, D. C. (2009). Quantification of five neuropsychological approaches to defining mild cognitive impairment. *American Journal of Geriatric Psychiatry*, *17*(5), 368-375. https://doi.org/10.1097/JGP.0b013e31819431d5

Lee, Y., Kim, J., Chon, D., Lee, K. E., Kim, J. H., Myeong, S., & Kim, S. (2018). The effects of frailty and cognitive impairment on 3-year mortality in older adults. *Maturitas*, *107*, 50-55. https://doi.org/10.1016/j.maturitas.2017.10.006

Lei, X. Y., Bai, C., Hong, J. P., & Liu, H. (2022). Long-term care insurance and the well-being of older adults and their families: Evidence from China. *Social Science & Medicine*, *296*, Article 114745. https://doi.org/10.1016/j.socscimed.2022.114745

Leone, A. J., Minutti-Meza, M., & Wasley, C. E. (2019). Influential observations and inference in accounting research. *Accounting Review*, *94*(6), 337-364. https://doi.org/10.2308/accr-52396

Li, C., Jin, S., Cao, X., Han, L., Sun, N., Allore, H., Hoogendijk, E. O., Xu, X., Feng, Q., Liu, X., & Liu, Z. (2022). Catastrophic health expenditure among Chinese adults living alone with cognitive impairment: Findings from the CHARLS. *BMC Geriatrics*, *22*(1), 640. https://doi.org/10.1186/s12877-022-03341-8

Lin, Z. E., Qian, Y. T., Gill, T. M., Hou, X. H., Allore, H., Chen, S. Q., & Chen, X. (2025). Absence of care among community-dwelling older adults with dementia and functional limitations. *Nature Aging*. https://doi.org/10.1038/s43587-025-00836-y

National Healthcare Security Administration of China, & Ministry of Finance of China. (2020). *Guiding opinions on expanding the pilot long-term care insurance*. Retrieved 2024-09-11 from https://www.gov.cn/gongbao/content/2020/content_5570107.htm

Pei, X. T., Yang, W., & Xu, M. M. (2024). Examining the impact of long-term care insurance on the care burden and labor market participation of informal carers: A quasi-experimental study in China. *Journals of Gerontology Series B-Psychological Sciences and Social Sciences*, *79*(5), Article gbae023. https://doi.org/10.1093/geronb/gbae023

Van den Berg, B., Brouwer, W., van Exel, J., Koopmanschap, M., van den Bos, G. A., & Rutten, F. (2006). Economic valuation of informal care: Lessons from the application of the opportunity costs and proxy good methods. *Social Science & Medicine*, *62*(4), 835-845. https://doi.org/10.1016/j.socscimed.2005.06.046

Van den Berg, B., Brouwer, W. B. F., & Koopmanschap, M. A. (2004). Economic valuation of informal care: An overview of methods and applications. *European Journal of Health Economics*, *5*(1), 36-45. https://doi.org/10.1007/s10198-003-0189-y

Wagstaff, A., & van Doorslaer, E. (2003). Catastrophe and impoverishment in paying for health care: With applications to Vietnam 1993-1998. *Health Economics*, *12*(11), 921-934. https://doi.org/10.1002/hec.776

World Bank. (2022). *Inflation, consumer prices (anual %) - China and Korea, Rep.* Retrieved 2024-07-14 from https://data.worldbank.org/indicator/FP.CPI.TOTL.ZG?locations=CN-KR&view=chart

Yang, J., Wang, S., & Du, S. (2018). Regional comparison and implications of China's long-term care insurance system (in Chinese). *Chinese Journal of Health Policy*, *11*(4), 1-7.

Yang, W., & Hu, B. (2022). Catastrophic health expenditure and mental health in the older Chinese population: The moderating role of Social Health Insurance. *Journals of Gerontology Series B-Psychological Sciences and Social Sciences*, *77*(1), 160-169. https://doi.org/10.1093/geronb/gbab130

Zhao, Y., Atun, R., Oldenburg, B., McPake, B., Tang, S., Mercer, S. W., Cowling, T. E., Sum, G., Qin, V. M., & Lee, J. T. (2020). Physical multimorbidity, health service use, and catastrophic health expenditure by socioeconomic groups in China: An analysis of population-based panel data. *Lancet Global Health*, *8*(6), E840-E849.

Zhou, W., & Dai, W. (2021). Shifting from fragmentation to integration: A systematic analysis of long-term care insurance policies in China. *International Journal of Integrated Care*, *21*(3), Article 11. https://doi.org/10.5334/ijic.5676
